# Ambient Air Pollution, Housing Context, and Birth Outcomes Among Wisconsin Mothers

**DOI:** 10.1007/s10995-024-03941-3

**Published:** 2024-06-07

**Authors:** Amy K. Fottrell, Marah A. Curtis, Fiona H. Weeks

**Affiliations:** 1 L. Douglas Wilder School of Public of Government and Public Affairs, Virginia Commonwealth University, 1808 Gordon Ave., Richmond, VA 23224 USA; 2 Sandra Rosenbaum School of Social Work, Institute for Research on Poverty, University of Wisconsin-Madison, 1350 University Ave, Madison, WI 53706 USA; 3 Department of Population Health Sciences, Center for Demography and Ecology, University of Wisconsin-Madison, 610 Walnut St., Madison, WI 53704 USA; 4 Division of Public Health, Wisconsin Department of Health Services, Madison, USA

**Keywords:** Air pollution, Housing and health, Birth outcomes, Birthweight

## Abstract

**Objectives:**

To assess the association between air pollution exposure and housing context during pregnancy and adverse birth outcomes.

**Methods:**

We linked air pollution data from the Environmental Protection Agency and housing data from the American Community Survey with birth records from Wisconsin counties over a 9-year period. We calculated average daily pregnancy exposure to fine particulate matter and ozone and modeled its relationship to preterm birth, low birthweight and NICU admission, adjusting for individual characteristics and housing context.

**Results:**

Ozone exposure and housing cost-burden had substantive and statistically significant negative associations with birthweight and gestational age, and positive associations with NICU admission, while a poor-quality housing environment had a significant negative effect on weeks of gestation. Fine particulate matter exposure had a negligible correlation with these outcomes.

**Conclusions for practice:**

An additional tenth of one part-per-million daily average exposure to ozone is associated with a 33 g decrease in birthweight. This decrease in birthweight is about the same size as the association of gestational diabetes (32 g), larger than the association of chronic hypertension (22 g), and about 40% the size of the effect of smoking during pregnancy on birthweight (84 g). Given the magnitudes of the associations with atmospheric ozone and adverse birth outcomes, reducing atmospheric ozone should be a public health priority. Inclusion of controls for housing cost-burden and poor-quality housing reduces the magnitude of the association with mothers who identify as Black, suggesting the importance of these structural factors in understanding adverse birth outcomes by race.

## Significance

**What is already known on this subject:**

It is well documented that poor birth outcomes are linked to experiences of social and economic disadvantage, however, environmental correlates of poor birth outcomes are under-researched. Existing research does not account for the ways in which environmental, sociodemographic, and structural factors could confound each other given the concentration of environmental pollutants in communities of color and low-income communities.

**What this study adds:**

In this paper, we account for individual sociodemographic characteristics, access to emergency infant healthcare, and neighborhood housing factors to clarify the independent association of air pollution on birth outcomes in Wisconsin across a nine-year period.

## Introduction

In Wisconsin in 2019, 10.1% of all babies were born preterm, and 7.6% were born with low birthweight (< 2500 g) (Centers for Disease Control and Prevention, 2022). Preterm birth and low birthweight contributed to about 16% of infant deaths in 2020 in the United States (Ely & Driscoll, 2022). Moreover, negative birth outcomes, including preterm birth and low birthweight, can affect the long-term health of infants even into adulthood including increasing risk for high blood pressure, obstructive lung disease, diabetes, and cognitive deficiencies (Luu et al., 2017). These health issues place burdens on families as well as on the broader healthcare system and economy (Petrou et al., 2001). It is well documented that poor birth outcomes are linked to experiences of social and economic disadvantage (Aizer & Currie, 2014; Blumenshine et al., 2010); however, environmental correlates of poor birth outcomes are under-researched. Although some recent papers consider the association of green space, (Laurent et al., 2019) housing quality, (Miranda et al., 2012; Suglia et al., 2011) and air pollution (Ibrahimou et al., 2014) on low birthweight across the United States, they do not consider the combined associations of multiple environmental factors.

Low-income communities are more likely to be exposed to air pollution, have less access to healthcare, experience pre-existing health conditions, and have lower quality housing stock (Hood, 2005; Lazar & Davenport, 2018). Existing research does not account for the ways in which environmental, sociodemographic, and structural factors could confound each other given the concentration of environmental pollutants in communities of color and low-income communities (Colmer et al., 2020). In this paper, we account for individual sociodemographic characteristics, access to emergency infant healthcare, and neighborhood housing factors to clarify the independent association of air pollution on birth outcomes in Wisconsin across a nine-year period.

### Air Pollution, Housing Context and Maternal and Child Health

In this study, we focus on fine particulate matter and ozone exposure as potential environmental hazards. Fine particulate matter (PM_2.5_) consists of small particulates from both natural and anthropogenic sources that are small enough to be inhaled and become lodged deep in the lungs. It can cause aggravation of asthma or existing lung disease, heart attacks, decreased lung function, and cancers (*Health and Environmental Effects of Particulate Matter (PM)*, 2022). Ozone is primarily a result of pollution from automobiles and industrial sources, especially in the summer months. Exposure to ozone can trigger airway inflammation and reduce lung function and can worsen bronchitis, emphysema, and asthma (*Health Effects of Ozone Pollution*, 2022).

Beyond the well-known cardiovascular and respiratory effects, prenatal exposure to PM_2.5_ and ozone can affect birth outcomes (Bekkar et al., 2020). Overall, it is estimated that exposure to air pollution during pregnancy results in a 10.8% increased risk of low birthweight and 11.5% increased risk of preterm birth. The mechanisms by which air pollution affects birth outcomes are hypothesized to be systemic inflammation, changes to maternal cardiac and respiratory function, placental inflammation, or direct fetal exposure to toxins, among other possibilities (Bekkar et al., 2020). Multiple pathways may operate simultaneously.

While air pollution has the same biological effect on a body regardless of sociodemographic characteristics, not all birthing people are equally vulnerable. Black birthing people and infants experience much greater levels of PM_2.5_ and ozone exposure than the rest of the population, followed by Asian and Latina moms and infants (Bekkar et al., 2020). Counties in the United States with the highest known ozone levels have significantly greater non-Hispanic Black populations than those with the lowest ozone levels (Miranda et al., 2011). Black Americans are more likely to live near roadways or industrial sites, to live in older, poorer quality homes, (Schulz et al., 2002) and to breathe air that is 38% more polluted than their white counterparts (Fleischman & Franklin, 2017). Disproportionate exposure to environmental pollution and poor housing stock contributes to higher rates of asthma, which is an independent risk factor for preterm birth, among Black women compared with their White peers. Pollution sources are often located in communities that also experience other forms of disadvantage, so controlling for potentially confounding neighborhood-level factors can help isolate the effects of air pollution. Finally, poor housing quality can be particularly dangerous for pregnant people. Studies have found that exposure to both poor-quality homes and crowding are associated with increased risk of low birthweight, small for gestational age, and birthweight percentile for gestational age (Harville & Rabito, 2018; Miranda et al., 2012). In communities with a limited supply of safe and affordable housing, housing cost-burden can force individuals to choose between paying rent, utilities, food, and medical care (Shaw, 2004), with accompanying stress also associated with adverse birth outcomes (Shapiro et al., 2013).

## Methods

We estimate the relationships between environmental factors and low birthweight, gestational age and NICU admission using a series of pooled regression models with county and year fixed effects to control for time-invariant factors impacting birth outcomes. We construct a dataset using 2011–2019 birth records from thirteen Wisconsin counties combined with EPA data on air pollution, Census Data on housing context, and U.S. Department of Health and Human Services data on infant emergency healthcare access. The thirteen counties were chosen because they house EPA air pollution monitors that report consistent, high-quality data on ozone and PM_2.5_. These counties represent 50% of the state population, include both rural and urban counties, and represented 54% of the total births in Wisconsin in 2016. We use residential census tracts and counties from the birth records to append pollution and housing measures. We excluded multiple births (twins, triplets, etc.) because they experience higher levels of poor birth outcomes than single births, all else equal (Atoof et al., 2015; Kurdi et al., 2004). Total singleton births during this period were 298,316. We excluded births to birthing people under the age of 16 (672 births) and over the age of 44 (379 births) because birthing people outside of typical child-bearing years are more likely to have poor birth outcomes (Carolan, 2013). We additionally excluded observations with birthweights under 500 g (151 births) and other births which are unlikely to be true live births based on the combination of birthweight and weeks of gestation (1,279 births). Approximately 7% of observations had one or more missing data points. We use data from the birth certificates to perform multiple imputation by chained equations (MICE) in order to handle missing data in individual characteristics. Missing data in ozone and PM_2.5_ data was extrapolated using an average of the nearest non-missing neighbors before and after the missing observation. Our final sample includes 295,835 singleton births to birthing people between 16 and 44 years old that occurred between 2011 and 2019 in thirteen counties in Wisconsin. All analyses were completed using StataMP17.

### Birth Outcomes

We model three birth outcomes as our dependent variables: weeks of gestation, standardized birthweight, and Neonatal Intensive Care Unit (NICU) admission.

Weeks of gestation is an obstetric estimate and preferrable to using a dichotomous preterm birth variable because gestational age in weeks is more strongly associated with infant health outcomes. Generally, outcomes are worse for infants born prior to 34 weeks, while late preterm birth (between 34 and 37 weeks) is more common. Additionally, early term births (37 or 38 weeks) tend to have higher rates of adverse outcomes than full term gestations (39 or 40 weeks) (Chawanpaiboon et al., 2019). Birth weights are normally distributed with variation by weeks of gestation, so we utilize the z-score methodology used by Oken et al (2003) to create a nearly continuous variable of birth weight for gestational age. Categorical divisions like “small for gestational age”, historically defined as those infants below the 10th percentile at each gestational age, may mask differences in risk for complications and later disease along the spectrum of birth weight (Oken et al., 2003). Oken’s continuous measure of birth weight relative to gestational age allows for a better understanding of the determinants of fetal development. NICU admission is a dichotomous variable determining whether an infant was admitted to the Neonatal Intensive Care Unit at the time of birth. Approximately half of the NICU admissions in our sample were infants who were either categorized as low birth weight or preterm. However, other conditions like Chronic Lung Disease (CLD) and jaundice can also be treated in the NICU. This measure accounts for other adverse outcomes in addition to low birth weight and preterm birth.

### Air Pollution

We use Air Quality System (AQS) data from the US Environmental Protection Agency (EPA) to create a measure of the cumulative amount of ozone and fine particulate matter that each pregnant person was exposed to over the course of pregnancy, using information from birth certificates including birthdate and weeks of gestation. Ozone is measured in parts per million (ppm), while PM_2.5_  is measured in micrograms per cubic meter, per the National Ambient Air Quality Standards (NAAQS) established by the EPA. NAAQS standards are intended to provide a threshold of pollutants that will protect both public health and the environment. For ozone, that threshold is measured by the annual fourth-highest daily maximum 8-hour concentration, averaged over 3 years, and should not exceed 0.070 parts per million by volume. PM_2.5_, has two standards. Either it should not exceed an annual mean of 9.0 micrograms per cubic meter of air as averaged over 3 years, or 35 micrograms per cubic meter of air as the 98th percentile averaged over 3 years (EPA, 2024). We then divide the cumulative exposure by the approximate number of days of gestation to create a measure of the average daily exposure during pregnancy, a proxy for the intensity of exposure. EPA AQS data is not granular enough to append it at the census tract level, but birthing people were matched to monitor data for the county in which they resided at the time of birth. While we cannot account for changes to residence during pregnancy, prior work on Wisconsin custodial mothers finds that Supplemental Nutrition Assistance Program (SNAP) recipients (proxy for low-income) are more likely to move multiple times a year, but national data suggests that moves are typically within the same area (Curtis & Warren, 2016).

### Housing Measures

We use two measures of the housing environment—the percent of households in a census tract that are cost-burdened and the percent of housing in a census tract that is poor-quality, using 2010–2019 American Community Survey (ACS) for a birthing person’s census tract. In the housing literature, a household is typically defined as being “cost-burdened” when they pay 30% or more of their annual household expenses toward housing costs (Colburn et al., 2024). We combine renter and homeowner measures from the ACS to create one variable representing the percentage of households in a tract that are housing cost-burdened. The U.S. Department of Housing and Urban Development’s (HUD) defines inadequate housing using data from the American Housing Survey on a nationally representative sample of housing units. Units are considered severely inadequate when they have no hot water, no running water, and no working toilet, exposed wires or electrical problems, electric shut off and no heat for 24 + hours, (U.S. Department of Housing and Urban Development, 2013). The measure of poor-quality housing we construct with data available in the ACS is the sum of the percent of homes in a census tract that have incomplete kitchens and the percent of homes that have incomplete plumbing capturing some important features of HUD’s definition. Both incomplete kitchens and incomplete plumbing are rare outcomes, but likely indicative that the housing stock in a census tract is, overall, of poorer quality. Inadequate housing is associated with poorer indoor air quality (Holden et al., 2023), if not directly with levels of ambient air pollution. At the very least, poor housing quality compounds existing environmental disadvantage so accounting for this factor is important.

### Structural Healthcare Factors

The number of NICU beds accounts for most of the variation in admission rates between hospitals; newborns born in hospitals with more NICU beds are more likely to be admitted to the NICU, particularly for milder conditions, while infants born in hospitals that have very few NICU beds are less likely to be admitted to the NICU even if needed (Haidari et al., 2021). To control for this, we obtained the number of NICU beds in each of the fourteen counties from the Area Health Resource File (AHRF) and calculated the number of NICU beds per 10,000 births in the county.

### Maternal and Child Characteristics

We adjust for maternal and child characteristics known to be associated with birthweight and gestational age, including maternal health conditions (diabetes and chronic or gestational hypertension), maternal age (grouped), maternal race (non-Hispanic White, non-Hispanic Black, Hispanic, and other), prenatal care adequacy (Kotelchuck index), maternal education, marital status, WIC enrollment, birth payer, and maternal smoking during pregnancy.

**Table 1:** Characteristics of births in select Wisconsin counties, 2011–2019

*N* = 295,835	Percent or Mean (SD)	Range
**Maternal Characteristics**		
*Maternal Race/Ethnicity*		
White, non-Hispanic (%)	0.62	
Black, non-Hispanic (%)	0.16	
Hispanic, any race (%)	0.12	
Asian, non-Hispanic (%)	0.06	
Multi-racial, non-Hispanic (%)	0.02	
American Indian/Alaska Native (%)	0.01	
Other/Unknown (%)	0.01	
Married (%)	0.61	
Maternal Age in Years	28.82 (5.5)	16–44
*Maternal Health Risks*		
Diabetes (%)	0.08	
Chronic Hypertension (%)	0.02	
Gestational Hypertension (%)	0.07	
Smoke During Pregnancy (%)	0.1	
Receives Medicaid (%)	0.38	
Receives WIC (%)	0.33	
**Healthcare Factors**		
Kotelchuck Index of Prenatal Care Adequacy		
Adequate Plus (%)	0.42	
Adequate (%)	0.37	
Intermediate (%)	0.05	
Inadequate (%)	0.16	
NICU Beds per 10,000 Births	14.35 (29.61)	0–1351.66
**Air Pollution Exposure**		
Average Ozone Exposure Per Day During Pregnancy (parts per 100,000)	0.42 (0.21)	0.55–1.16
PM Exposure Per Week Pregnancy (ug/m3)	11.45 (9.45)	0.53–40.78
**Census Housing Context**		
Housing Cost Burdened (%)	0.35 (0.12)	0.09–0.94
Low Quality Housing (%)	0.037 (0.05)	0–0.59
**Birth Outcomes**		
Birth Weight in Grams	3338.67 (558.44)	500–5940
Gestation in Weeks	38.68 (1.83)	22–44
NICU Admission (%)	0.08	

**Table 2:** Racial composition of births, ambient air pollution, and housing context of select Wisconsin Counties 2011–2019

County	N	% Total Sample	% Black	% White	% Hispanic	Mean Daily PM Exposure (ug/m3)	Mean Daily Ozone Exposure (ppm)	% Cost-Burdened	% Low Quality Housing
Ashland	1,212	0.4%	0.08%	80.8%	3.14%	1.706	0.029	30.1%	13.5%
Brown	28,041	9.36%	4.41%	70.9%	12.5%	8.331	0.034	28.9%	2.03%
Dane	51,007	17.0%	7.82%	71.5%	9.00%	10.810	0.033	31.9%	1.75%
Dodge	6,764	2.26%	0.71%	89.9%	7.41%	5.281	0.033	28.6%	2.46%
Eau Claire	9,678	3.23%	1.04%	85.8%	2.79%	3.164	0.030	29.5%	4.42%
Forest	866	0.29%	0.46%	67.1%	2.90%	2.509	0.034	25.8%	15.0%
Grant	3,831	1.28%	1.07%	94.1%	2.77%	4.306	-	25.7%	5.11%
Kenosha	14,767	4.93%	10.7%	65.0%	19.1%	4.919	0.060	37.3%	4.17%
La Crosse	10,160	3.39%	1.68%	84.5%	2.13%	4.866	0.032	27.0%	1.73%
Milwaukee	114,507	38.2%	34.3%	36.6%	18.3%	18.467	0.065	42.8%	5.83%
Outagamie	18,315	6.11%	1.29%	83.7%	5.46%	5.94	0.033	24.9%	2.49%
Sauk	6,308	2.1%	0.57%	87.1%	8.18%	5.184	0.034	30.5%	3.39%
Taylor	1,889	0.63%	0.21%	94.5%	3.07%	2.609	0.032	27.5%	11.2%
Waukesha	32,349	10.8%	1.62%	84.1%	6.65%	4.973	0.031	27.3%	1.13%

Selected counties are included due to availability of ambient air pollution data and include Ashland, Brown, Dane, Dodge, Eau Claire, Forest, Grant, Kenosha, La Crosse, Milwaukee, Outagamie, Sauk, Taylor, Waukesha

**Table 3:** Adjusted regression results for select birth outcomes, 2011–2019 Births in 14 Wisconsin counties

	Birth Weight^1^ (*N* = 219,375) Point estimate: − 0.011	Weeks Gestation^1^ (*N* = 219,375) Point estimate: 38.68	NICU Admission^2^ (*N* = 219,069) Point Estimate: 0.083
**Environmental Factors**			
*Air Pollution*			
PM Exposure (ug/m3)	0.000 (0.000)	-0.003^***^ (0.001)	1.005^***^ (0.002)
Ozone Exposure (parts per 100,000)	-0.072^**^ (0.023)	-0.219^***^ (0.041)	1.333^***^ (0.111)
**Housing Context**			
Cost Burden	-0.102^***^ (0.026)	-0.132^**^ (0.046)	1.226^*^ (0.118)
Low Quality Housing	-0.081 (0.047)	-0.358^***^ (0.083)	1.436^*^ (0.237)
**Maternal Characteristics**			
*Race/Ethnicity*			
White	0.172^***^ (0.008)	0.187^***^ (0.013)	1.017 (0.029)
Black	-0.018^*^ (0.009)	-0.234^***^ (0.016)	1.108^**^ (0.036)
Hispanic	0.127^***^ (0.009)	0.079^***^ (0.016)	0.955 (0.033)
Married	0.030^***^ (0.006)	0.095^***^ (0.010)	0.786^***^ (0.017)
*Age*			
16–19	-0.093^***^ (0.011)	0.061^***^ (0.019)	1.136^***^ (0.043)
20–24	-0.042^***^ (0.007)	0.028^*^ (0.012)	0.994 (0.024)
30–34	0.036^***^ (0.006)	-0.049^***^ (0.010)	1.039 (0.022)
35–39	0.041^***^ (0.007)	-0.152^***^ (0.013)	1.152^***^ (0.030)
40+	0.037^*^ (0.015)	-0.269^***^ (0.026)	1.166^***^ (0.059)
*Education*			
Less than High School	-0.016^*^ (0.008)	0.036^**^ (0.014)	0.964 (0.026)
More than High School	0.010 (0.006)	0.069^***^ (0.010)	0.950^*^ (0.021)
*Maternal Health Risks*			
Diabetes	0.129^***^ (0.008)	-0.369^***^ (0.014)	1.520^***^ (0.039)
Chronic Hypertension	-0.052^***^ (0.013)	-1.098^***^ (0.024)	2.582^***^ (0.090)
Gestational Diabetes	-0.047^***^ (0.009)	-0.968^***^ (0.015)	2.460^***^ (0.059)
Smoking during pregnancy	-0.157^***^ (0.008)	-0.302^***^ (0.014)	1.591^***^ (0.039)
Receives WIC	0.002 (0.006)	0.084^***^ (0.010)	0.888^***^ (0.019)
Receives Medicaid	-0.001 (0.006)	-0.076^***^ (0.011)	1.129^***^ (0.025)
**Healthcare Factors**			
*Kotelchuck Index*			
Adequate Plus	0.007 (0.005)	-0.799^***^ (0.009)	1.860^***^ (0.037)
Intermediate	-0.014 (0.010)	0.023 (0.017)	1.332^***^ (0.051)
Inadequate	-0.024^***^ (0.006)	-0.443^***^ (0.011)	1.867^***^ (0.045)
NICU Beds per 10,000 Births			1.016^***^ (0.001)
**Child Characteristics**			
Birth Weight Z-Score			0.992 (0.008)
Male	0.162^***^ (0.004)	-0.055^***^ (0.007)	1.245^***^ (0.020)

^1^Birth weight and weeks gestation models are linear regression models. The birth weight model uses a transformation to the z-score of birth weights based on work by Oken et al. (2003); coefficients are represented in z-scores

^2^NICU admission is a logistic regression with results presented as odds ratios.

* *p* < 0.05, ** *p* < 0.01, *** *p* < 0.001

The reference category for age is 25–29. The reference category for education is high school. The reference category for Kotelchuck Index is adequate

### Ethical Approval

This secondary analysis was determined to be exempt from human subjects research oversight by the Institutional Review Board of the University of Wisconsin - Madison.

## Results

Sample characteristics can be found in Table 1. Our sample somewhat overrepresents Black (16%) and Hispanic (12%) birthing people compared to all people giving birth in Wisconsin because of the exclusion of many rural counties without EPA monitors. Our sample aligns closely with the state as a whole in terms of maternal age, education, marriage, and health risk factor variables. Several included rural counties have no NICU beds, while Dane County and Milwaukee County have 84 and 118 beds for every 10,000 residents, respectively.

Mean exposure to both ozone and PM_2.5_ fall underneath the NAAQS attainment thresholds, but over the course of our study, parts of Kenosha, Milwaukee, and Waukesha counties have been nonattainment areas, with levels of pollution exceeding limits set by the EPA. On average, the birthing people in our sample live in census tracts where about 4% of homes are of poor quality and 35% of households are cost-burdened, with census tracts ranging from about 9–94% housing cost-burdened, exhibiting large amounts of variation within counties.

Birthweights for observations included in the sample range from 500 to 5940 g at birth. The length of pregnancy for the samples ranges from 22 to 44 weeks, with a mean of 38.7 weeks. Slightly over 8% of infants in our sample were admitted to the NICU.

Table 2 examines the geographic variation in births, race and ethnicity, pollution exposure and housing environments by county. Births were geographically distributed similar to general population distribution in the state, with about 38% of the sample in Milwaukee County and 17% in Dane County, while some rural counties represent less than 1% of births. There is wide variation in the typical daily exposure to PM_2.5_ and ozone by maternal county of residence. Most counties experience daily ozone levels between 0.024 ppm and 0.034 ppm, but Kenosha and Milwaukee counties experience levels about twice that high. Most counties registered average daily PM_2.5_ levels under 6 ug/m3; however, Milwaukee, Dane, and Brown counties averaged 18.4 ug/m3, 10.81 ug/m3, and 8.32 ug/m3 respectively. In line with previous studies about racial disparities in exposure, 97.6% of Black and 86.9% of Hispanic birthing people in the sample live in Milwaukee, Kenosha, Dane, or Brown counties, where they are exposed to higher levels of air pollutants. About 59% of white birthing people in the sample live in those same counties. Similarly, Milwaukee and Kenosha counties have higher percentages of cost-burdened households compared to the other counties in the sample. However, small, rural, and mostly white counties experience disproportionately high levels of low-quality housing stock, which is reflective of national trends (Housing Assistance Council, 2023).

### Regression Analysis

Table 3 provides regression results examining the association of air pollution, housing cost and quality on birthweight, weeks of gestation and NICU admission controlling for maternal characteristics and healthcare factors. Ozone exposure has a negative and statistically significant association with all three birth outcomes, with an additional 0.1 ppm average daily exposure associated with a 33 g decrease in birthweight, a slightly shorter gestation period, and a 26% higher likelihood of admission to the NICU. PM_2.5_ had a statistically significant and negative association with length of gestation and NICU admission, but the magnitude of the coefficients is negligible.

Every 1% increase in the number of people in a census tract who are housing cost-burdened is associated with a 60 g reduction in birthweight and a reduction in weeks of gestation of about one day. The results show a significant 41% increase in NICU admission among infants of birthing people living in a high housing cost-burden environment. Living in an environment with poor housing quality was associated with a decrease in length of gestation (2.5 days) but did not have a statistically significant association with NICU admission or birthweight. Maternal characteristics, generally, were associated with the birth outcomes consistent with previous research. Black birthing people in the sample had lower birthweight infants, fewer weeks of gestation and a significantly higher incidence of NICU admission than either white or Hispanic birthing people, while married birthing people had higher birthweights, more weeks of gestation and a lower likelihood of NICU admission than unmarried birthing people.

Maternal health risks, NICU beds, Medicaid receipt, and prenatal care quality generally had the expected effects based on the literature. Because pregnant people who receive prenatal care that is “Adequate Plus” typically receive additional care because they have one or more risk factors, it is also unsurprising that birthing people in this category had shorter gestation periods and were 84% more likely to have infants that were admitted to NICU, compared with birthing people with adequate care. Receipt of WIC benefits seems to have slight protective effects, with people receiving WIC during pregnancy having a small but significant increase in weeks of gestation and a slightly lower chance of NICU admission.

## Discussion

Consistent with previous studies, we found that increased ozone exposure during pregnancy was associated with increased incidence of negative birth outcomes; namely, lower birthweight, shorter gestational age, and higher rates of NICU admission. Importantly, we found that neighborhood cost-burden and low-quality housing also had a negative impact on these outcomes, independent of the effect of air pollution. These findings suggest that the negative effects of structural disadvantage are compounded by each other; moreover, they suggest that eliminating or ameliorating some of these structural disadvantages could begin to chip away at the persistent geographic and racial inequities in birth outcomes.

Our results are limited by the level of geography of air pollution data; appending data at the county level does not account for differences within counties due to topography and distance from point sources. We also cannot account for the mobility of people throughout their pregnancy or exposures to pollution in places other than the home environment. These constraints may have contributed to the negligible effects of PM_2.5_ on birth outcomes because ozone data is seasonal and therefore more variation is detectable even within a large geography, and further research could benefit from more granular air pollution data. Similarly, housing data from the ACS provides information about the community housing environment that surrounds a person during pregnancy but cannot provide information about a person’s individual housing experience. Our study shows that housing environments that induce community-level stress can potentially aggravate poor birth outcomes regardless of an individual’s housing circumstances.

Moreover, we excluded births to people under 16 and over 44 because they are more likely to have poor birth outcomes. However, pregnant people under 16 in particular are more likely to be Black, native, or Hispanic and are additionally more likely to live in communities with higher levels of both PM_2.5_ and ozone, as well as communities with higher levels of cost-burden. People in the sample over age 44 were more likely to live in communities with higher levels of PM_2.5_. Therefore, our results may underestimate the full effects of ambient air pollution among individuals already at higher risk for poor outcomes.

In aggregate, our results show that multiple environmental and community factors can compound to produce negative health effects. Many birthing people reside in communities that experience multi-layered and interwoven forms of distress, and researchers should continue to parse these outcomes in order to effectively tailor public policy across sectors. This research underscores the need for a Health in All Policies approach to policy-making with tools like Health Impact Assessments (Lock, 2000), which take into account all existing environmental factors.

The hospitalization costs for low birthweight and preterm infants in the United States was estimated at $5.8 billion annually in 2001 (Russell et al., 2007). This estimate does not include the family psychological strain in caring for these infants, the increased risk of infant death, long-term costs associated with increased care needed by some infants across their lifetimes nor the later-life health outcomes associated with being born too early, or too small. Additionally, the burdens of low birthweight and preterm birth disproportionately impact birthing people of color, (Osterman et al., 2022) and contribute to racial inequities in health across the life course. A focus on amenable environmental factors is a fruitful area for policymakers to consider to improve population health and save public dollars. Maternal and child health advocates should explore collaborations with advocates on housing and environmental policy to increase their impact on eliminating persistent inequities in birth outcomes.

## Data Availability

The data used for this research were requested from the Wisconsin Department of Health Services Vital Records Office.
